# Severely Painful and Pruritic Forearm Rash: A Case of Caterpillar Envenomation in South Florida

**DOI:** 10.5811/cpcem.1679

**Published:** 2024-03-26

**Authors:** Cody M. Autrey, Stephanie A. Martinez, Michael Remaly, Eric Boccio

**Affiliations:** * Florida International University, Herbert Wertheim College of Medicine, Miami, Florida; † Memorial Healthcare System, Department of Emergency Medicine, Hollywood, Florida

**Keywords:** *Megalopyge opercularis*, *asp caterpillar*, *caterpillar envenomation*, *lepidopterism; case report*

## Abstract

**Introduction:**

The asp caterpillar (*Megalopyge opercularis*) is endemic to the southeastern United States, with most sightings in Florida, Texas, and Louisiana. A few hundred caterpillar envenomations are reported annually with most cases occurring in July–November. Asp caterpillars have hollow spines along their backs that contain venom. Contact with these spines is what produces the characteristic “sting” resulting in contact dermatitis and a localized hypersensitivity reaction collectively referred to as lepidopterism. Symptoms of lepidopterism may include severe burning pain, pruritis, edema, nausea, vomiting, abdominal pain, and headache. Symptoms are often self limited, and treatment should focus on expedited removal of implanted spines and aggressive symptom management.

**Case Report:**

We present the case of a patient presenting to the emergency department (ED) with acute-onset severe left forearm pain with associated pruritic rash incurred while working in a retail store. Initial therapeutic management included administration of analgesics, antihistamines, and steroids. After obtaining a comprehensive history and consulting with the Poison Control Center, we suspected an asp caterpillar envenomation. Following extraction of the caterpillar spines with silk tape, the patient’s symptoms improved. After a period of observation in the ED, the patient was discharged home without any known sequelae.

**Conclusion:**

Although asp caterpillars typically inhabit trees and foliage, human exposure to the caterpillar may occur in developed environments. Effective history-taking, prompt communication with toxicologic experts, and complete removal of intact spines are essential for early identification and effective clinical management of asp caterpillar envenomation.

Population Health Research Capsule What do we already know about this clinical entity?

*Asp caterpillar (*Megalopyge opercularis*) envenomation is an unusual cause of a severely painful rash affecting patients in the southeastern United States.*

What makes this presentation of disease reportable?

*While exposure risks are greatest outdoors near trees and foliage, caterpillar stings may also occur in commercial buildings and residential settings.*

What is the major learning point?

*Comprehensive history-taking, heightened clinical suspicion, interdisciplinary collaboration, and effective therapeutic management are necessary to treat envenomation.*

How might this improve emergency medicine practice?

*Awareness of exposure risks and symptoms of erucism and recognition of the characteristic erythematous rash will facilitate timely diagnosis and proper treatment.*

## INTRODUCTION

The asp caterpillar (*Megalopyge opercularis*), also known as the woolly slug caterpillar or puss caterpillar, is the larva form of the southern flannel moth. It has a wide geographic distribution stretching from Maryland to Mexico and is endemic to the southeastern United States with most sightings in Florida, Texas, and Louisiana.^1^ Peak months of asp caterpillar reports occur between July–November.^2^ The asp caterpillar is typically 2.5–4 centimeters (cm) in length, and its hairs range in color from gray to yellow-brown giving it its characteristic woolly appearance. Within the dense hair coat are hidden spines called setae, which are hollow and contain a poison gland. Contact may cause the setae to fracture and inject venom, resulting in both toxic effects and type IV hypersensitivity/immunoglobulin E-mediated reactions. The asp caterpillar sting is reported to be the most potent of all caterpillar stings in the United States.^3^

While most caterpillar envenomations are benign and do not necessitate medical evaluation, various clinical sequelae have been described. The asp caterpillar sting causes a characteristic grid-like, hemorrhagic eruption, and the rash is associated with excruciating pain and irritation, puncture wounds, pruritis, and edema.^4^ Sting reactions include erucism (localized urticarial dermatitis), lepidopterism (skin and systemic reactions), ophthalmia nodosa (ocular inflammatory reaction), and lonomism (a potentially life-threatening bleeding diathesis).^5^ Systemic toxicity may manifest as lymphadenopathy, headache, nausea, vomiting, abdominal pain, and pseudoappendicitis.^6^^–^^8^ The reaction is often self-limiting; management should focus on expeditious removal of any remaining implanted setae and aggressive symptom management. Albeit rare, there have been several reports of anaphylactic shock resulting from caterpillar envenomation.^9^

We report an unusual cause of acute, sudden onset, severe left forearm pain and pruritis in an adult male who was at work in a retail store in South Florida. Although these caterpillars typically inhabit trees and foliage, this case highlights that human exposure to asp caterpillars may occur in developed environments. Effective history-taking, heightened clinical suspicion, prompt communication with toxicologic experts, and complete removal of intact spines are essential for early identification and effective clinical management of asp caterpillar envenomation.

## CASE REPORT

A 20-year-old male with no known past medical history or allergies presented to the emergency department (ED) complaining of acute, sudden onset, left forearm pain and rash. The patient was working at an eyeglasses and contacts store and stated that the pain started when he knelt to lift something up from ground level. In doing so, he placed his left elbow on his left thigh. He immediately felt a stinging sensation in his left elbow with severe 10/10 pain radiating down the extremity to the left forearm and hand. Shortly afterward he noticed the affected area had become erythematous, thus he presented to the ED for an emergent medical evaluation. Pertinent negatives included absence of fever, chills, cough, shortness of breath, chest pain, nausea, vomiting, diarrhea, urinary complaints, back pain, and headache.

**Image 1.**
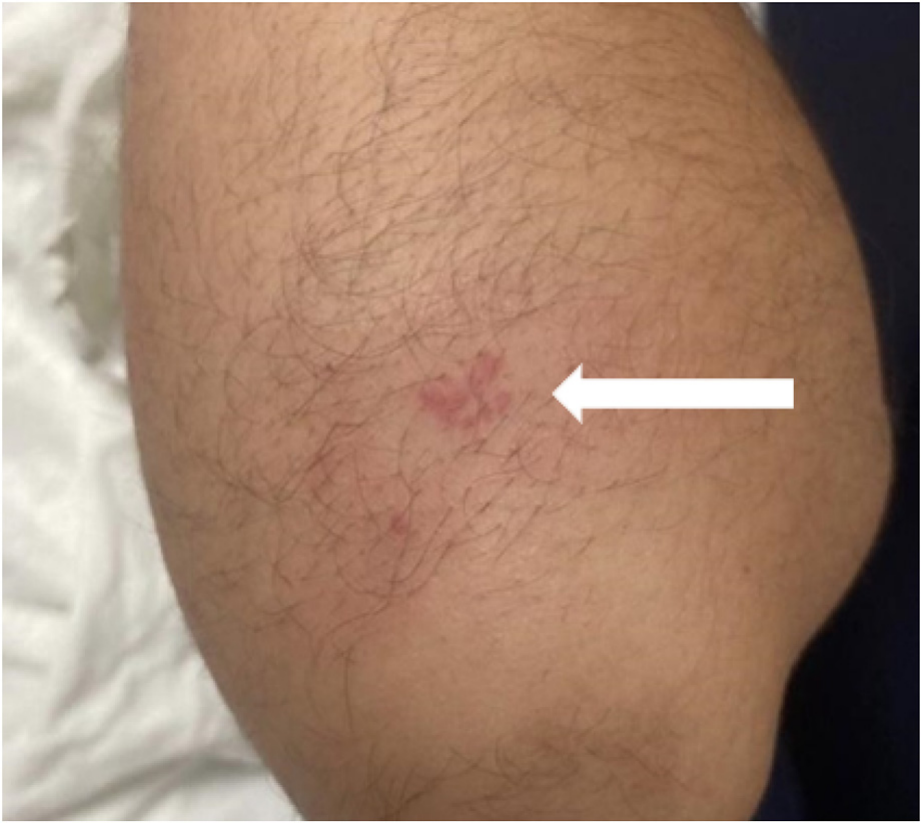
Erythematous spots appearing at the site of asp caterpillar envenomation on the posterior proximal forearm (arrow).

The patient arrived hypertensive with initial blood pressure measuring 130/91 millimeters of mercury. Triage vital signs were otherwise normal. On physical examination, the patient appeared anxious. The primary survey of airway, breathing, and circulation was unremarkable. Focused examination of the affected region demonstrated an approximately 1 × 2-cm area of erythema and edema over the proximal posterior forearm with an associated grid-like pattern of raised urticaria. The rash was localized and did not exhibit desquamation (Image 1).

**Image 2.**
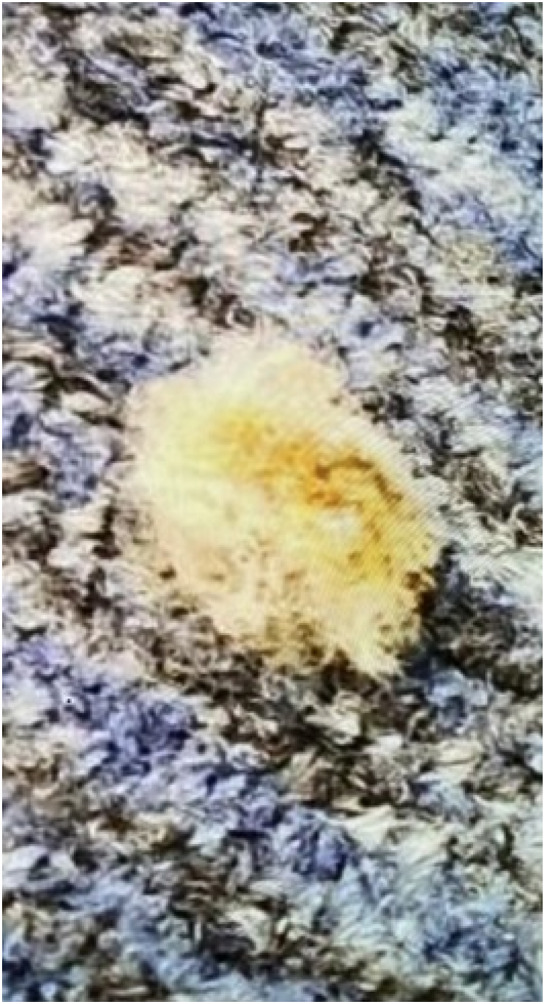
Photo from smartphone of an asp caterpillar (*Megalopyge opercularis*) on the carpeted floor of an eyeglass and contact lens store.

The left upper extremity was otherwise neurovascularly intact with a palpable radial pulse and capillary refill less than three seconds. Assessment of motor function and active and passive range of motion at the elbow was normal. Laboratory assessment and imaging were determined to be of no utility and were not ordered. Upon further inquiry, the patient mentioned that his arm may have contacted a caterpillar, which was found on the ground at the site where his pain first began. A photo of the caterpillar was eventually obtained and provided on the patient’s smartphone (Image 2).

The emergency physician called the Poison Control Center (PCC) hotline, which confirmed that the symptoms were likely the result of an asp caterpillar envenomation. Of note, the expert at the PCC mentioned that the presentation was particularly unusual given that it occurred in a commercial building devoid of trees and foliage. Other diagnoses considered were allergic reaction, insect bite/sting, contact dermatitis, cellulitis, and traumatic injury.

Therapeutic management focused on aggressive symptom management. Due to concern for a developing hypersensitivity reaction, dexamethasone 8 milligrams (mg) intravenous (IV), acetaminophen-codeine 300-30 mg per os (PO), famotidine 20 mg IV, ketorolac 30 mg IV, and diphenhydramine 25 mg IV were administered. Upon further inspection of the affected area, several spines were visualized penetrating the patient’s skin. Silk tape was applied to the affected area and carefully removed, thus stripping away the offending spines. The patient’s pain rapidly improved. The patient received an additional 25 micrograms of fentanyl IV after reassessment an hour later. He underwent a period of observation in the ED and was discharged with prescriptions for methylprednisolone 4 mg PO and oxycodone-acetaminophen 5–325 mg PO. Attempts to contact him after discharge to arrange follow-up and a wound check were unsuccessful. There were no subsequent patient encounters documented in the electronic health record.

## DISCUSSION

We describe a case report of a 20-year-old male with no known past medical history or allergies who presented to the ED with severe localized pain, erythema, and swelling of the left forearm. A focused assessment revealed an erythematous, swollen, and tender area containing several raised erythematous lesions in a grid-like pattern. Upon closer inspection, several spines were visualized remaining in the skin. Effective history-taking, recognition of the characteristic rash, timely involvement of an interdisciplinary team that included toxicologic experts at the PCC and species identification using available technologies facilitated timely diagnosis and appropriate clinical management.

Typically, asp caterpillar stings occur in outdoor settings when an individual unknowingly brushes against the caterpillar or the caterpillar falls from a tree where it normally resides. This case challenges the conventional exposure risks to asp caterpillars, describing an encounter that occurred indoors in an area devoid of any surrounding trees or foliage. It is possible that the caterpillar was carried into the store while attached to the patient’s clothing, within infested goods, or on a delivery package. The absence of detailed entomological investigations limited our ability to definitively ascertain how the asp caterpillar may have entered the store.

Interdisciplinary team collaboration played an essential role in this case. Effective communication between healthcare professionals and local toxicologic experts at the PCC was instrumental in establishing the diagnosis and guiding therapeutic management. The content knowledge regarding regional insect patterns and venomous species possessed by toxicologic experts aided in species identification and reliable prognostication.

Due to the paucity of reports in the literature and presumed underreporting in the clinical environment, the prevalence of atypical and severe presentations of asp caterpillar envenomation is unknown. The therapeutic management of asp caterpillar envenomation is variable. Usually benign and self-limiting, management should focus on removing the offending agent and symptom management. Administration of analgesics can be supplemented with topical and injected local anesthetics.^10^^,^^11^ An analysis of asp caterpillar stings reported to Texas Poison Centers found that numerous treatments such as dilution, irrigation and washing, antihistamines, steroids, and antibiotics have been effectively used.^4^ While analgesics, antihistamines, and steroids are the staples of caterpillar envenomation, no consensus guidelines exist for its management in the ED setting.

## CONCLUSION

Although asp caterpillars typically inhabit trees and foliage, human exposure may occur in developed environments. Caterpillar envenomation is most likely underreported and mistreated due to the lack of patient and clinician knowledge, lack of detail in the history of present illness, and common mimics of erucism. Effective history-taking, heightened clinical suspicion, prompt communication with toxicologic experts, and complete removal of intact spines are essential for early identification and effective clinical management of asp caterpillar envenomation. The relatively high prevalence of asp caterpillar envenomation justifies future study of its venom, development of a potential antivenom, and guidelines for clinical management.
